# Biomarker signatures as predictors of future impulsivity in schizophrenia: a multi-center study

**DOI:** 10.3389/fpsyt.2025.1620131

**Published:** 2025-09-29

**Authors:** Siqi Liu, Yixiao Chen, Lei Zhang, Xu Zhang, Jiali Min, Yaqin Yang, Manru Li, Zheya Cai, Yanwei Sun, Jiayi Wang, Zhihao Chen, Hui Li, Fazhan Chen, Jiaojiao Hou, Ruyi Shui, Guoquan Zhou, Enzhao Zhu

**Affiliations:** ^1^ Shanghai Putuo District Mental Health Center, Shanghai, China; ^2^ Shanghai Tongji Hospital, Tongji Hospital of Tongji University, Shanghai, China; ^3^ School of Medicine, Tongji University, Shanghai, China; ^4^ Shanghai Yangpu District Mental Health Center, Shanghai, China; ^5^ East China University of Science and Technology, Shanghai, China; ^6^ Shanghai Key Laboratory of Psychotic Disorders, Shanghai Mental Health Center, Shanghai Jiaotong University School of Medicine, Shanghai, China; ^7^ Clinical Research Center for Mental Disorders, Shanghai Pudong New Area Mental Health Center, School of Medicine, Chinese-German Institute of Mental Health, Tongji University, Shanghai, China; ^8^ University Clinic of Child and Adolescent Psychiatry, Psychosomatics and Psychotherapy, Rheinisch-Westfälische Technische Hochschule (RWTH) Aachen University, Aachen, Germany

**Keywords:** schizophrenia, impulsivity, machine learning, biomarkers, causal inference

## Abstract

**Introduction:**

While clinical scales for impulsivity assessment in psychiatric settings are widely used, evidence linking laboratory biomarkers to impulsivity remains limited. This study evaluated the prognostic value of routinely collected biomarkers for future impulsivity risk and developed a machine learning–based prediction model.

**Methods:**

We analyzed data from 1,496 first-admission schizophrenia (SCZ) patients across four specialized psychiatric hospitals (2016–2023). A total of 99 features, including 91 routinely tested biomarker measurements, four treatment-related indicators, and four demographic or psychometric variables, were evaluated. Impulsivity was assessed using the Impulsive Behavior Risk Assessment Scale within one week of admission. Five machine learning models were trained with 10-fold cross-validation (n=993) and externally validated in an independent cohort (n=503). Model performance was assessed using the area under the receiver operating characteristic curve (AUROC), and biomarker importance was evaluated using SHapley Additive exPlanations (SHAP).

**Results:**

Of 1,496 SCZ patients, 882 (59.0%) exhibited high impulsivity. CatBoost outperformed other models, achieving an AUROC of 0.749 in cross-validation and 0.719 in external testing. SHAP values identified key biomarkers, revealing heterogeneous response patterns for uric acid (UA), globulin (GLO), apolipoprotein E (APOE), and others. Combining biomarkers with clinical data improved prediction, increasing AUROC from 0.652 to 0.749 in cross-validation and from 0.655 to 0.721 in external testing. Subgroup analyses revealed sex-specific patterns, with exploratory analysis suggesting sex-modified relationships between UA and impulsivity.

**Discussion:**

These findings highlight the utility of routine biomarkers for early identification of high-risk individuals with SCZ and suggest the importance of incorporating sex-specific factors in predictive modeling.

## Introduction

Impulsivity is a core feature of multiple psychiatric disorders and represents a major public health challenge (1). Among them, schizophrenia (SCZ) exhibits the most severe and disruptive forms, marked by sudden, uncontrolled acts of violence or self-harm. Individuals with SCZ not only show elevated levels of impulsivity but are also at increased risk of victimization (2, 3). Epidemiological studies report a 49–68% higher risk of violent behavior in this population compared to the general public, underscoring the clinical relevance of impulsivity in SCZ (4, 5). This heightened impulsivity contributes to poorer clinical outcomes, prolonged hospitalization, and substantial healthcare burden. In psychiatric inpatient settings, it also poses a persistent threat to both staff and patients (6). Traditional interventions have shown limited efficacy in preventing impulsivity in SCZ (7). As a result, early identification and targeted prevention of impulsivity are paramount, representing not only a critical step toward improving clinical outcomes but also an urgent public health priority.

In China, the Impulsive Behavior Risk Assessment Scale (IBRAS) (8), a composite of the Modified Overt Aggression Scale and Impulsivity Screening-10, is widely used to screen hospitalized patients with SCZ. While it facilitates risk identification, its reliance on self-report and observer ratings may limit early proactive intervention (9). While tools like the IBRAS are widely adopted and clinically useful, they are primarily designed for contemporaneous risk monitoring during hospitalization, often failing to capture biological signals that precede overt behavioral escalation (10, 11). Developing a robust, data-driven prognostic model could overcome these limitations and support early, individualized intervention. However, most existing studies have focused on cross-sectional associations with current impulsivity (12, 13), rather than longitudinal prediction of future risk. Moreover, many are limited by small sample sizes, single-center designs (14), or insufficient control of confounding factors (15). Additionally, the association between sex and impulsivity in SCZ remains controversial, with studies reporting inconsistent findings (16, 17). Leveraging a large, multicenter real-world dataset, we employed propensity score matching (PSM) to control for confounding and reduce selection bias. This study aimed to elucidate the association between sex and future impulsivity in SCZ, evaluate the predictive value of routine biomarkers, and develop a clinically applicable risk model using machine learning. Early identification of impulsivity risk during the initial days of hospitalization can inform individualized treatment planning, proactive ward management, and preventive interventions (18). Such early warning systems may complement weekly clinical assessments (e.g., IBRAS) by identifying high-risk individuals prior to routine evaluations, offering timely insights that bridge the critical period immediately following admission, thereby potentially improving patient outcomes and ward safety (19).

## Methods

### Ethics

We first examined the association between routinely collected biomarkers and impulsivity. Subsequently, we applied machine learning algorithms to evaluate their predictive performance and develop a clinically applicable risk model. This study was approved by the Ethics Committee of Shanghai Putuo District Mental Health Center (approval number: M202409) and conducted in accordance with the principles of the Declaration of Helsinki. Reporting followed the Strengthening the Reporting of Observational Studies in Epidemiology (STROBE) guidelines (20).

### Data sources

Data were obtained from four psychiatric institutions in Shanghai: Putuo District Mental Health Center, Tongji University Mental Health Center, Changning District Mental Health Center, and the Shanghai Mental Health Center, which is one of China’s four National Medical Centers for Mental Diseases. The dataset comprises comprehensive real-world electronic health records, including admission details, diagnostic codes, psychometric assessments, medical prescriptions, laboratory results, and structured risk evaluations from both inpatient and outpatient settings. This multicenter design enhances the generalizability of findings and reflects the clinical profile of psychiatric inpatients across China.

### Study participants

**Figure 1:**
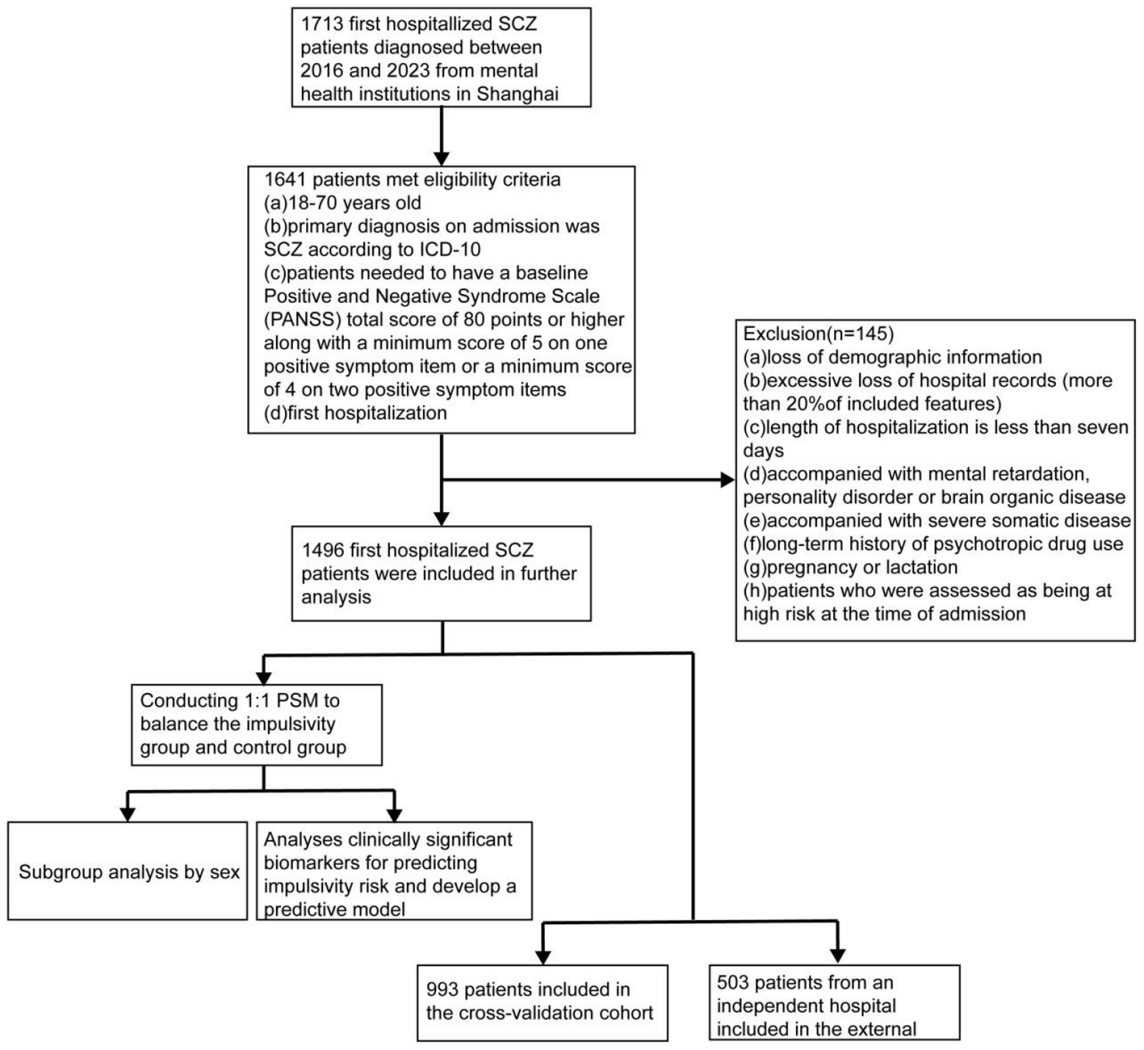
Procedure. SCZ, schizophrenia; PSM, propensity score matching.

This retrospective cohort study included detailed and comprehensive medical records of psychiatric patients hospitalized from January 2016 to March 2023. The eligibility criteria included (a) 18–70 years old, (b) diagnosis on admission was SCZ based on ICD-10, (c) patients needed to have a baseline Positive and Negative Syndrome Scale (PANSS) total score of 80 points or higher, along with a minimum score of 5 on one positive symptom item or a minimum score of 4 on two positive symptom items (21), (d) first hospitalization, (e) resident in China. Exclusion criteria included (a) loss of demographic information, (b) excessive loss of hospital records (more than 20% of included features), (c) length of hospitalization is less than seven days, (d) accompanied with mental retardation, personality disorder or brain organic disease, (e) accompanied with severe somatic disease, (f) long-term history of psychotropic drug use, and (g) pregnancy or lactation. In addition, patients who were assessed as being at high risk at the time of admission were also excluded. The overall patients’ inclusion process is presented in **Figure 1**. The analysis was carried out between October 2023 and December 2024.

### Temporal procedures

Each patient was assigned a unique hospitalization identifier, enabling longitudinal tracking across admissions. The first traceable admission was designated as the index hospitalization, from which demographic information was extracted. Medication records and electroconvulsive therapy (ECT) data were obtained from electronic medical orders. Laboratory and auxiliary examinations included complete blood count, urinalysis, liver and renal function panels, immune markers, and other routinely assessed clinical indicators. In total, 99 features were assessed, including 91 biomarkers. Detailed definitions and variable descriptions are provided in **Supplementary Table S1**.

Laboratory and clinical examination data were obtained on the day of admission or within the preceding week, as some patients may have completed these assessments in outpatient settings prior to hospitalization. Given the delayed onset of psychiatric medication efficacy, medication and ECT records were extracted from one month prior to admission up to the day of admission. To account for cross-institutional treatment, prescription data were retrieved across all participating hospitals within this window. In addition, for patients who exhibited impulsivity during hospitalization, biomarker data within one week following the escalated impulsivity event were collected to enable time trend analysis.

### Grouping criteria

After meeting the inclusion criteria, patients were classified into two groups based on their IBRAS assessments within the first seven days of hospitalization. The impulsivity group included those with a maximum IBRAS total score ≥ 5 during this period, indicating high risk (22). The control group comprised patients who had IBRAS total scores < 5 at all available assessments within the same timeframe and did not exhibit any escalation in risk thereafter. Patients with missing IBRAS data or ambiguous risk trajectories were excluded to ensure diagnostic consistency. To ensure temporal precedence, only impulsivity events that occurred after the baseline biomarker collection (i.e., after admission) were included for model training. This design ensures that predictors temporally precede the outcome, supporting a prognostic modeling framework.

### Statistical analyses

To enhance transparency and reproducibility, all code used for data preprocessing, model development, and analysis has been made publicly available at: https://github.com/huahaoXie/SCZ/tree/master. Initial data curation was conducted using PostgreSQL 4.2, and all statistical analyses were performed in R (v4.1.3). Continuous variables were summarized as means with standardized deviations (SDs), and categorical variables as counts and percentages (%). Normality was assessed using the Anderson–Darling test. Depending on distribution, continuous variables were compared using the Welch *t*-test or Mann–Whitney *U* test; categorical variables were analyzed using the *χ²* test or Fisher’s exact test, as appropriate. Multiple comparisons were corrected using the Holm–Bonferroni method. All P values were two-sided, with significance defined as *P* < 0.05. In the data cleaning process, to prevent sample bias and ensure accurate results, data with missing values exceeding 30% were deleted. For data with missing values below 30%, imputation using chained equations was performed separately on training, testing, and independent cohorts. To mitigate potential confounding arising from demographic and clinical differences, PSM was performed to balance the impulsivity and control groups on age, sex, and treatment exposure. High-risk patients were matched 1:1 to controls using the nearest-neighbor algorithm, ensuring equal group sizes and covariate balance in the matched cohort. The effectiveness of matching was evaluated using inter-group comparisons and standardized mean differences (SMDs).

Missing values were handled using multiple imputation by chained equations, incorporating all available measurements and participant-level characteristics (23). Feature selection using least absolute shrinkage and selection operator (LASSO) logistic regression, which penalizes less informative features by shrinking their coefficients to zero, was performed exclusively within the cross-validation cohort. This strategy was adopted to prevent data leakage and to ensure an unbiased assessment of model performance. The odds ratios (OR) were obtained using random effects logistic regression models, in which the source hospital of the data was treated as a random effect (24). Confidence intervals (CIs) for changes before and after the onset of impulsivity were estimated using paired Welch’s *t*-test or Mann–Whitney *U* test for continuous variables, and bootstrapping for categorical variables, as appropriate. Bootstrapping was performed with 1,000 resamples, applying bias-corrected and accelerated adjustments. Subgroup analyses by sex were performed for both regression models and time trend evaluations. A *post hoc* sample size calculation was conducted assuming a conservative anticipated model R² of 0.10, an outcome prevalence of 49%, and 31 selected candidate predictors (25). The minimum required sample size was estimated to be approximately 900 patients to ensure a shrinkage factor ≥ 0.9 and optimism < 5%. Our final dataset included 1,496 patients, including 882 impulsivity events, which exceeds this threshold and supports robust model development and external validation.

### Predictive modeling

To evaluate the predictive utility of biomarkers for future impulsivity, we developed and compared a series of machine learning and deep learning models. All models were implemented in Python 3.8 using the following packages: XGBoost (v1.7.2), CatBoost (v1.2.7), LightGBM (v3.3.3), and scikit-learn (v1.1.3). Ten algorithms were applied: XGBoost, CatBoost, AdaBoost, LightGBM, gradient boosting machine (GBM), random forest (RF), multilayer perceptron (MLP), Bayesian network (BN), support vector machine (SVM), and logistic regression (LR). These approaches have been widely used in clinical prediction and demonstrate reliable performance across diverse healthcare applications (26, 27).

The Shanghai Mental Health Center was randomly designated as the external testing cohort, with the remaining sites used for model training and validation. The models were trained based on 10-fold cross-validation repeated ten times in the training cohort for the best replicability. Hyperparameter tuning was performed via randomized search (n=100 iterations) during cross-validation. For tree-based models (CatBoost, XGBoost, LightGBM, GBM, RF), we optimized parameters such as maximum tree depth (range: 3–10), learning rate (0.01–0.1), number of estimators (100–1000), L2 regularization (λ = 0–10), and minimum child weight (1–10). In CatBoost, categorical feature encoding was handled internally using ordered boosting to mitigate overfitting. For SVM, radial basis function kernels were used, with tuning of the regularization parameter C (0.1–10) and kernel coefficient γ (0.01–1). For LR, we tuned the inverse regularization strength (C) over a log-uniform grid from 0.001 to 100 and selected the optimal penalty (L1 vs. L2). For BN, we applied constraint-based structure learning (PC algorithm) with significance thresholds ranging from 0.01 to 0.2, followed by maximum likelihood estimation of parameters. Network structure stability was evaluated via repeated subsampling to ensure consistent edge selection. For the MLP, we tested network architectures with 1–3 hidden layers and 64–256 neurons per layer. ReLU activation, dropout (0.1–0.5), and L2 regularization (α = 1e–5 to 1e–3) were tuned jointly. Models were trained using the Adam optimizer with early stopping based on validation loss, using a patience of 10 epochs. Batch size was fixed at 64, and the maximum number of epochs was set to 100. Class imbalance was addressed using oversampling of the minority class within the training data. All preprocessing was performed separately within each cross-validation loop to avoid data leakage.

Model performance was primarily assessed using the area under the receiver operating characteristic curve (AUROC), supplemented by F1 score, area under the precision–recall curve (AUPRC), sensitivity, and specificity to capture both discrimination and class-specific performance. A blank model containing no predictors was included as a reference. To evaluate the incremental predictive value of biomarkers, model performance was compared with and without biomarker features. Model performance metrics were compared using Nadeau and Bengio’ s corrected resampled *t*-test (28), which accounts for the statistical dependence introduced by repeated sampling. Feature importance was quantified using gain-based (F1 score improvement) metrics and visualized via SHAP values (29) to facilitate model interpretability.

## Results

### Patients and matching

A total of 1,496 patients with first-time admission for schizophrenia met the inclusion criteria. Specifically, participants were enrolled from four centers as follows: Putuo District Mental Health Center (*n*=230), Tongji University Mental Health Center (*n*=540), Changning District Mental Health Center (*n*=223), and the Shanghai Mental Health Center (*n*=503). The latter served as the external validation cohort. Among them, 882 (59.0%) developed high impulsivity within one week of hospitalization, while the remaining 614 served as controls. Male patients were more likely to exhibit high impulsivity compared with females (54.4% vs 45.6%; *P*=0.001; **Supplementary Figure S2**).

After propensity score matching, 547 high-impulsivity patients were matched 1:1 to controls. Post-matching, baseline characteristics were balanced: the mean age was 46.3 ± 13.7 years in the impulsivity group and 48.8 ± 14.0 years in controls, with male proportions of 54.7% and 51.0%, respectively. Covariate balance was evaluated using inter-group comparisons and SMDs (**Supplementary Table S2**, **Supplementary Figure S1**). Detailed patient characteristics are presented in **Supplementary Table S2**.

### Data imputation

In data preprocessing, three biomarkers with missingness >30% were excluded. For the remaining 91 biomarkers, 13 had missing values <30% and were retained for analysis. Missing values were imputed using multiple imputation by chained equations. To avoid data leakage, imputation was performed separately within the training, internal testing, and external validation cohorts. In total, 16,473 (12.1%) of all biomarker feature datapoints were imputed.

### The association of biomarkers and future impulsivity risk

LASSO regression was applied to 91 clinical biomarkers, yielding 31 variables with non-zero coefficients as potential predictors of impulsivity. Corresponding regression coefficients are provided in **Supplementary Table S3**.

**Figure 2:**
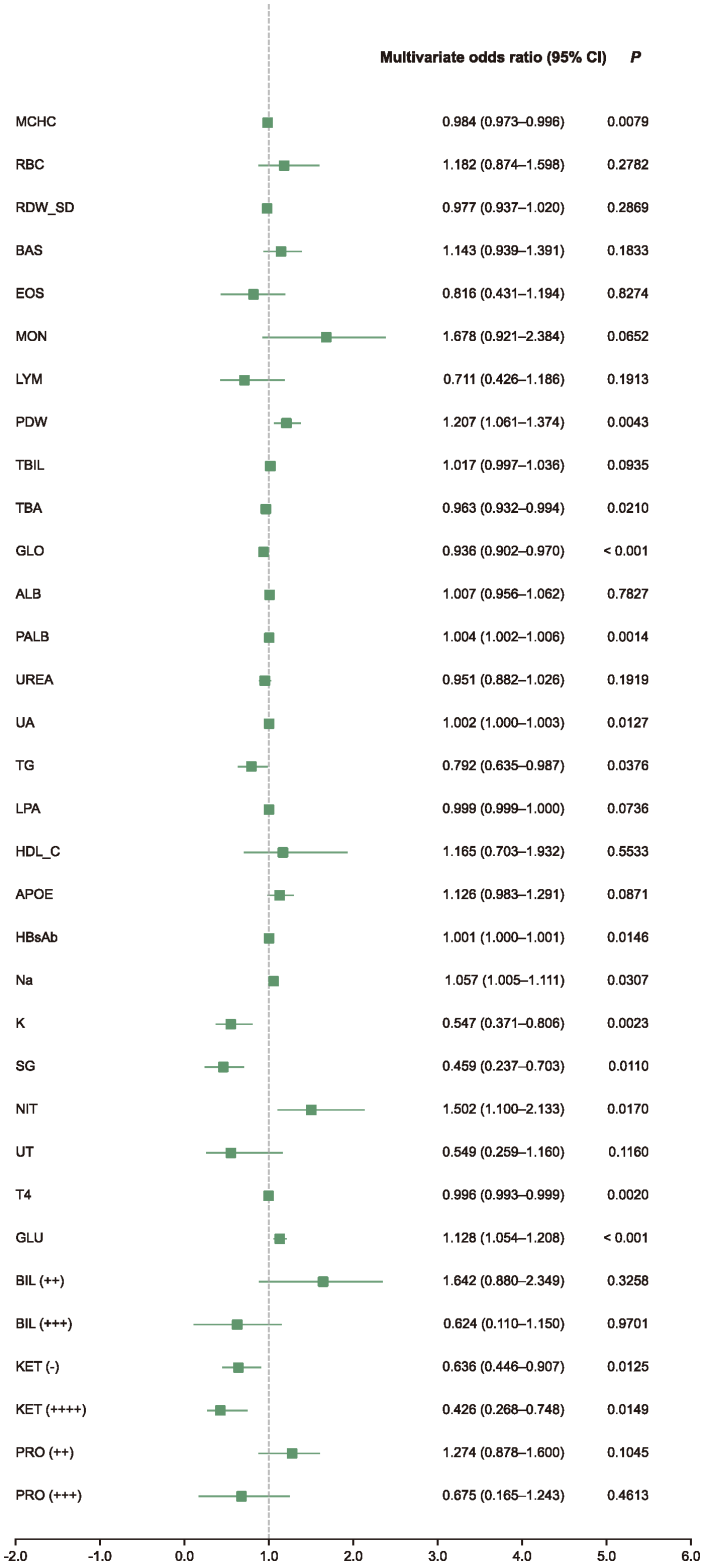
Multivariate analysis forest plot. This forest plot shows multivariate-adjusted odds ratios (ORs) and 95% confidence intervals (CIs) for selected biomarkers retained after LASSO feature selection. The analysis was conducted using logistic regression adjusted for study site as a random effect. Biomarkers with OR > 1 were associated with increased impulsivity risk, while those with OR < 1 were associated with decreased risk.

Subsequently, univariate and multivariate logistic regression analyses were performed on the selected biomarkers, identifying several with statistically significant associations with impulsivity (**Supplementary Table S4**). The high-risk indicators identified by ORs were mean platelet width (PDW), prealbumin (PALB), uric acid (UA), hepatitis B virus surface antibody (HBsAb), natrium (Na), urinary nitrite (NIT), fasting glucose (GLU), urine ketones (KET) negative; While the low-risk indicators included mean corpuscular hemoglobin concentration (MCHC), total bile acid (TBA), globulin (GLO), kalium (K), tetraiodothyronine (T4) (**Figure 2**).

**Figure 3:**
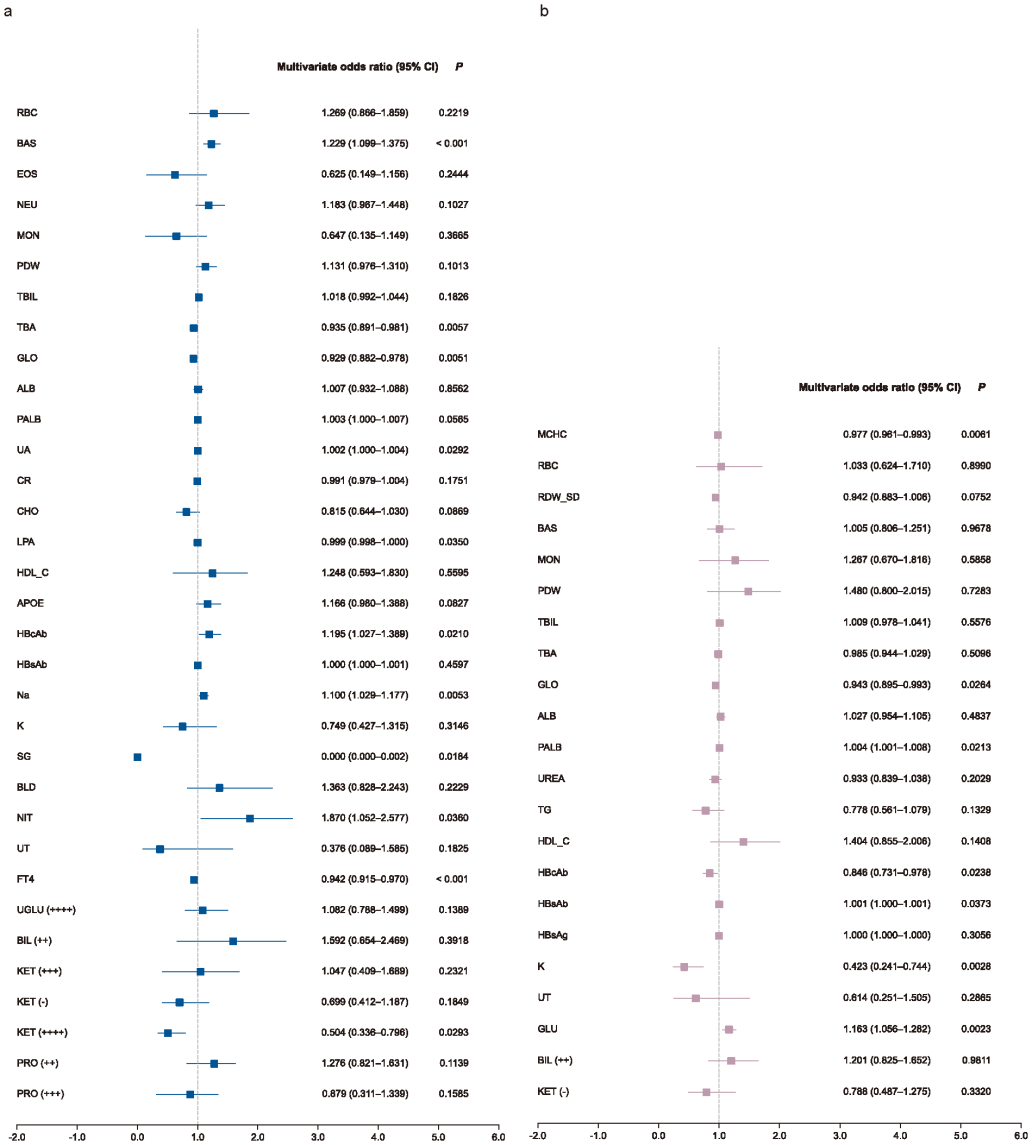
Subgroup multivariate analysis forest plot. Forest plots show multivariate-adjusted odds ratios (ORs) and 95% confidence intervals (CIs) for selected biomarkers in male **(a)** and female **(b)** patients. Logistic regression models were adjusted for study site as a random effect. Biomarkers with OR > 1 indicate increased risk; those with OR < 1 indicate protective associations.

Subgroup analyses revealed both overlapping and sex-specific patterns. Several indicators, such as UA, GLO, Na, NIT, and HBcAb, showed consistent associations across subgroups, aligning with the overall trend. In male group, additional predictors included basophil count (BAS), lipoprotein (LPA), free thyroxine (FT4), and KET positivity (**Figure 3A**). In female group, MCHC, PALB, HBsAb, K, and GLU emerged as significant (**Figure 3B**). Furthermore, exploratory interaction analysis suggested a potential modifying effect of sex on the relationship between UA and impulsivity risk (*P*=0.012; **Supplementary Table S4**).

**Figure 4:**
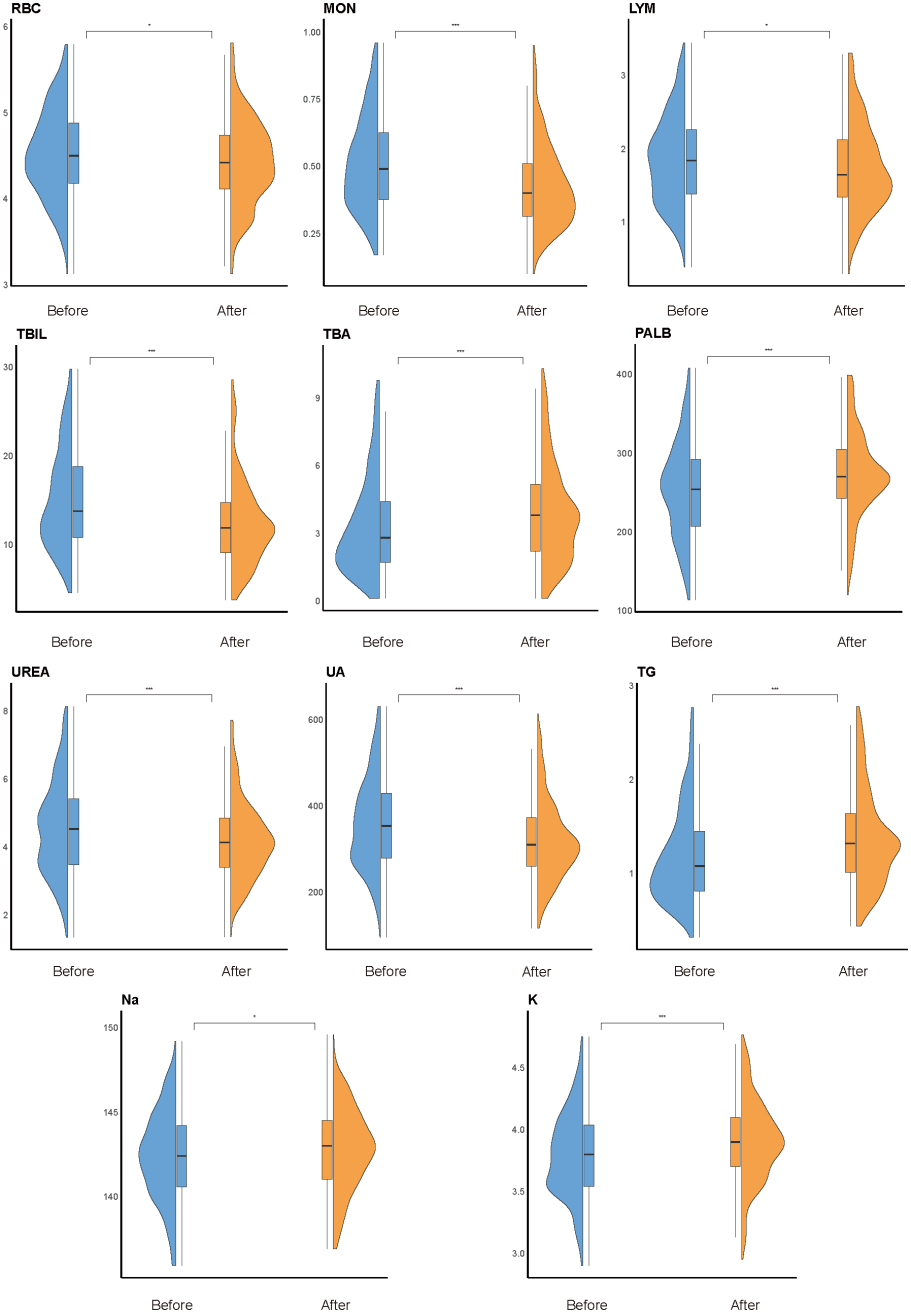
Overall time-trend analysis. Violin plots show longitudinal changes in the distribution of key biomarkers within the impulsivity group, comparing levels within one week of admission (“before”) and 180 days after escalation (“after”). Statistical significance was determined using paired Welch’s *t*-tests; *P < 0.05, **P < 0.01, ***P < 0.001. Box plots within violins represent the median and interquartile range.

**Figure 5:**
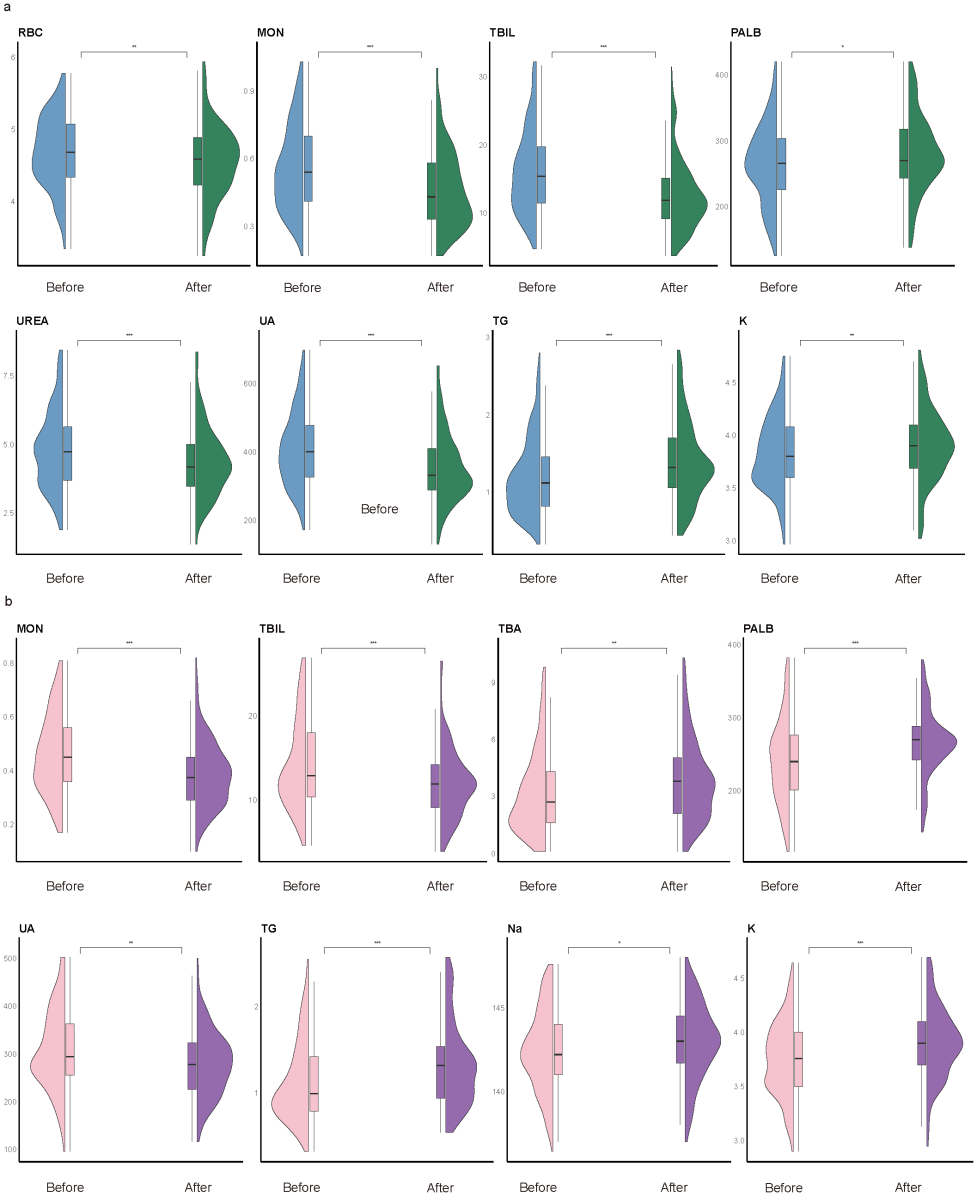
Subgroup time-trend analysis. Violin plots illustrate longitudinal changes in biomarker levels from within one week of admission (“before”) to 180 days after impulsivity escalation (“after”), stratified by sex. **(a)** Male subgroup. Significant temporal changes were observed in red blood cell count (RBC), monocytes (MON), total bilirubin (TBIL), prealbumin (PALB), uric acid (UA), triglycerides (TG), potassium (K), and urea (UREA). **(b)** Female subgroup. Significant variation was found in MON, TBIL, total bile acid (TBA), PALB, UA, TG, sodium (Na), and K. Notably, MON, TBIL, PALB, UA, TG, and K exhibited consistent time-dependent changes in both sexes. Statistical comparisons were conducted using paired Welch’s *t*-tests; *P < 0.05, **P < 0.01, ***P < 0.001. Box plots indicate medians and interquartile ranges.

To explore the temporal dynamics of biomarkers associated with impulsivity, we analyzed follow-up data from the impulsivity group at seven days after risk escalation (**Supplementary Table S5**). Several biomarkers previously identified as significant in the regression analysis, including UA, PALB, TBA, Na, and K, exhibited notable longitudinal changes (**Figure 4**). Additional indicators with significant time trends included red blood cell count (RBC), basophils (BAS), eosinophils (EOS), monocytes (MON), lymphocytes (LYM), total bilirubin (TBIL), urea nitrogen (UREA), triglycerides (TG), and apolipoprotein E (APOE). Subgroup analyses revealed sex-specific trends: RBC and UREA showed more pronounced changes in male group (**Figure 5A**), whereas TBA and Na exhibited greater variability in female group (**Figure 5B**). Several indicators, including MON, TBIL, PALB, UA, TG, and K, demonstrated consistent time-dependent changes in both sexes, further supporting their potential relevance in risk monitoring.

### Model performance and feature importance

**Figure 6:**
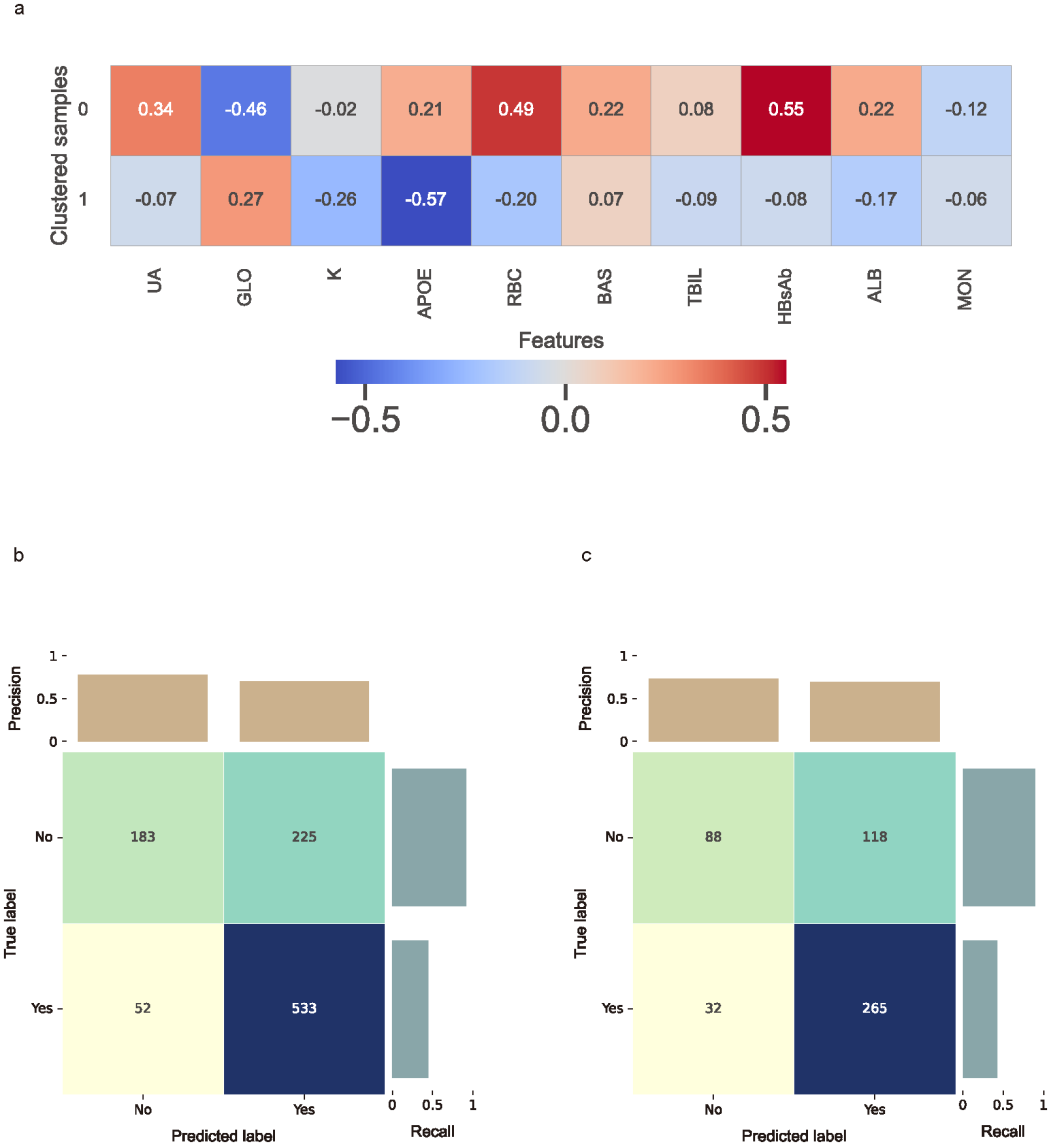
Clustered SHAP values heatmap and the confusion matrix. **(a)** Clustered SHAP (Shapley Additive Explanations) heatmap showing distinct feature attribution patterns across patient subgroups, based on top 10 biomarkers. Clustering was performed using K-means based on individual SHAP value profiles. Color intensity reflects mean SHAP values per biomarker within each cluster, with red indicating increased predicted risk and blue indicating reduced contribution. **(b)** Confusion matrix for the cross-validation cohort. **(c)** Confusion matrix for the external testing cohort. Confusion matrices present true and predicted labels for impulsivity, with darker cells representing higher counts.

CatBoost outperformed other models in both the cross-validation and external testing cohorts, achieving the highest AUROC of 0.749 (95% CI 0.714–0.783) and 0.719 (95% CI 0.664–0.767), respectively. It also showed superior F1 scores (0.794, 95% CI 0.768–0.824) and sensitivity (0.910, 95% CI 0.886–0.934), demonstrating its robust predictive performance across cohorts (**Table 1**, **Figures 6B, C**).

**Table 1:** Model performance.

Cross-validation cohort					
Model	AUROC	F1	AUPRC	Sensitivity	Specificity
Logreg	0.722 (0.696–0.748)	0.753 (0.735–0.771)	0.751 (0.711–0.785)	0.803 (0.777–0.821)	0.680 (0.657–0.705)
RF	0.743 (0.711–0.780)	0.788 (0.762–0.814)	0.763 (0.720–0.810)	0.894 (0.865–0.921)	0.729 (0.697–0.765)
SVM	0.659 (0.614–0.693)	0.749 (0.720–0.774)	0.703 (0.647–0.753)	0.889 (0.863–0.915)	0.654 (0.616–0.694)
BN	0.654 (0.618–0.691)	0.622 (0.581–0.658)	0.708 (0.659–0.753)	0.550 (0.508–0.592)	0.615 (0.579–0.647)
GBM	0.738 (0.706–0.770)	0.775 (0.748–0.802)	0.765 (0.722–0.805)	0.874 (0.844–0.902)	0.708 (0.672–0.743)
LightGBM	0.747 (0.712–0.789)	0.775 (0.746–0.803)	0.776 (0.734–0.819)	0.844 (0.813–0.875)	0.708 (0.676–0.746)
AdaBoost	0.713 (0.677–0.750)	0.778 (0.749–0.805)	0.743 (0.698–0.787)	0.940 (0.922–0.964)	0.727 (0.686–0.768)
CatBoost	**0.749 (0.714–0.783)**	**0.794 (0.768–0.824)**	**0.778 (0.736–0.819)**	0.910 (0.886–0.934)	0.742 (0.711–0.775)
XGBoost	0.730 (0.691–0.766)	0.783 (0.756–0.798)	0.765 (0.717–0.814)	**0.985 (0.976–0.997)**	**0.782 (0.753–0.814)**
MLP	0.701 (0.659–0.734)	0.748 (0.719–0.776)	0.736 (0.688–0.782)	0.804 (0.770–0.832)	0.672 (0.637–0.707)

External testing cohort					
Model	AUROC	F1	AUPRC	Sensitivity	Specificity
Logreg	0.675 (0.635–0.728)	0.734 (0.697–0.782)	0.706 (0.666–0.791)	0.782 (0.748–0.820)	0.662 (0.629–0.713)
RF	0.720 (0.668–0.769)	0.779 (0.738–0.816)	0.741 (0.679–0.803)	0.887 (0.843–0.925)	0.711 (0.659–0.764)
SVM	0.625 (0.570–0.679)	0.737 (0.693–0.775)	0.674 (0.597–0.736)	0.882 (0.840–0.918)	0.622 (0.556–0.684)
BN	0.659 (0.598–0.717)	0.685 (0.637–0.738)	0.715 (0.642–0.776)	0.650 (0.595–0.722)	0.643 (0.597–0.687)
GBM	0.697 (0.636–0.754)	0.758 (0.716–0.793)	0.734 (0.679–0.795)	0.869 (0.827–0.911)	0.673 (0.611–0.725)
LightGBM	0.708 (0.662–0.759)	0.766 (0.727–0.800)	0.746 (0.685–0.805)	0.843 (0.801–0.887)	0.692 (0.645–0.737)
AdaBoost	0.669 (0.604–0.728)	0.749 (0.706–0.787)	0.697 (0.626–0.765)	0.900 (0.856–0.939)	0.649 (0.580–0.710)
CatBoost	**0.719 (0.664–0.767)**	0.781 (0.740–0.812)	**0.751 (0.687–0.807)**	0.894 (0.854–0.924)	0.714 (0.663–0.756)
XGBoost	0.696 (0.646–0.750)	**0.787 (0.745–0.822)**	0.723 (0.663–0.790)	**0.976 (0.956–0.992)**	**0.775 (0.714–0.816)**
MLP	0.675 (0.625–0.725)	0.735 (0.695–0.778)	0.714 (0.641–0.780)	0.810 (0.760–0.854)	0.644 (0.596–0.693)

This table presents the performance metrics of different machine learning models across cross-validation and external testing sets. Reported values are point estimates and 95% confidence intervals obtained using bootstrap with 1000 iterations. AUROC, Area Under the Receiver Operating Characteristic Curve; F1, F1 score; AUPRC, Area Under the Precision-Recall Curve; LogReg, Logistic Regression; RF, Random Forest; SVM, Support Vector Machine; BN, Bayesian Network; GBM, Gradient Boosting Machine; MLP, Multilayer Perceptron. Bolded font indicates the best metric across models within the same set.

In the CatBoost model, the ten most important features, ranked by average gain across the cross-validated cohort, included TBIL, ALB, T4, UREA, RBC, APOE, K, HBsAb, GLO, and UA (**Supplementary Figure S3A**). In the external testing cohort, SHAP analysis identified UA, GLO, K, APOE, RBC, BAS, TBIL, HBsAb, ALB, and MON as the top contributors to model predictions (**Supplementary Figure S3B**).

To capture group-level feature contributions while accounting for potential interactions, individual SHAP values were clustered using the K-means algorithm. The optimal number of clusters was determined to be two based on the silhouette score (**Supplementary Figure S4**). The clustered SHAP heatmap (**Figure 6A**) revealed heterogeneous response patterns for UA, GLO, APOE, RBC, TBIL, HBsAb and ALB in relation to predicted impulsivity.

### Added benefits of biomarkers

**Figure 7:**
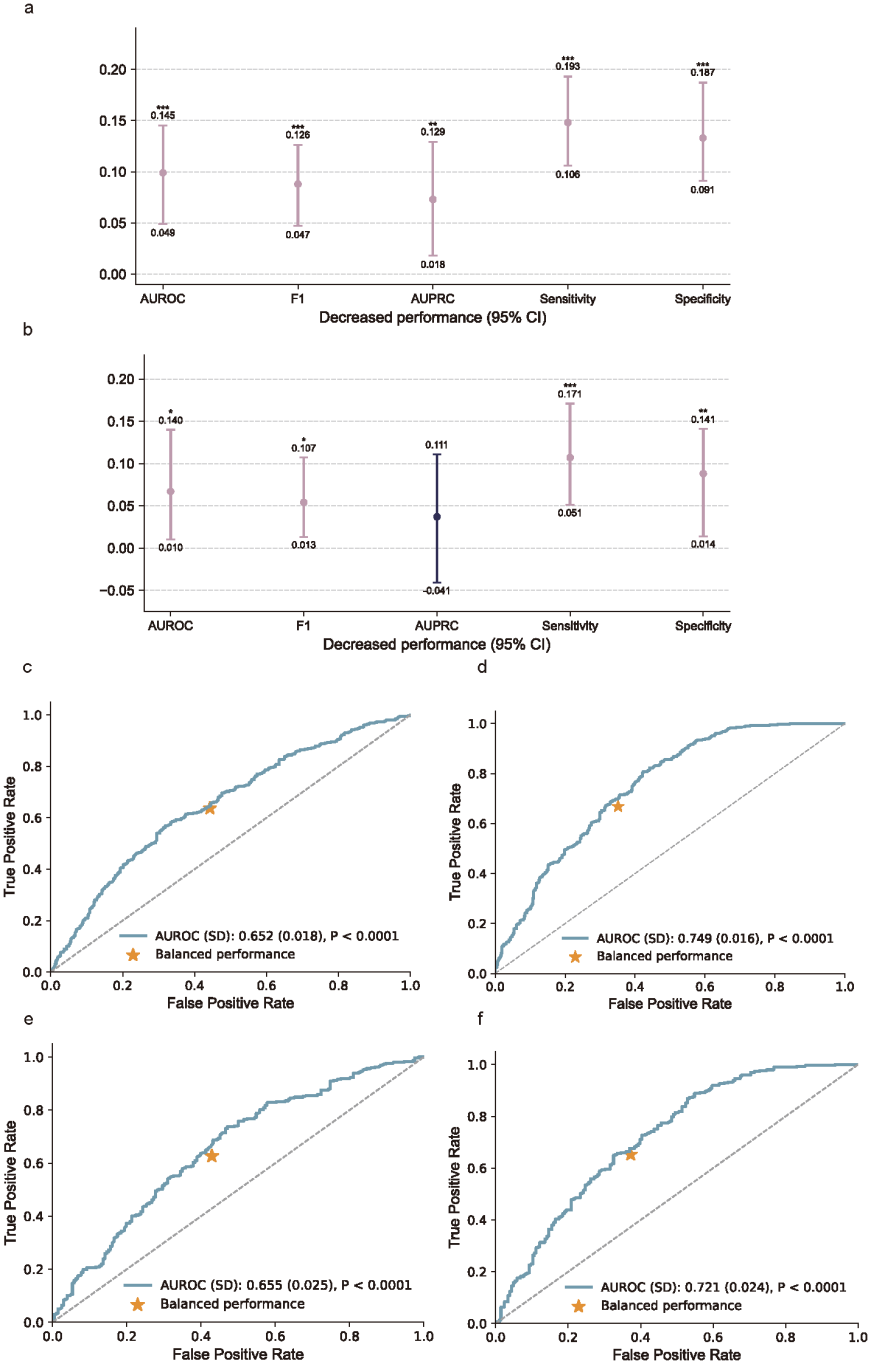
Added benefits of biomarker measurements and the receiver operating characteristic curves (ROC). **(a)** Performance gain from incorporating biomarker features in the cross-validation cohort, assessed using AUROC, F1 score, AUPRC, sensitivity, and specificity. **(b)** Performance gain in the external testing cohort. Bars indicate absolute differences in model performance between models with and without biomarkers, with 95% confidence intervals. *P* values were calculated using Nadeau and Bengio’s corrected resampled t-test; *P < 0.05, **P < 0.01, ***P < 0.001. **(c–f)**, Receiver operating characteristic (ROC) curves comparing models without **(c, e)** and with **(d, f)** biomarker inputs in the cross-validation cohort **(c, d)** and external testing cohort **(e, f)**. Inclusion of biomarkers substantially improved model discrimination, as indicated by increased AUROC values and more favorable balance points.

Integrating biomarkers with the baseline clinical data significantly improved model performance. In the cross-validation cohort, the combined model showed a higher AUROC (difference = 0.087; 95% CI, 0.047–0.126), F1, AUPRC, sensitivity, and specificity compared to the model without biomarkers (*P* < 0.001; **Figure 7A**). In the external testing cohort, the addition of biomarkers also led to a significant improvement in AUROC, F1 score, sensitivity, and specificity (**Figure 7B**).

By comparing the results of the cross-validation cohort and the external testing cohort, we aim to enhance the assessment of the model’s generalization performance. In the cross-validation cohort, the ROC curve for the model without biomarkers (**Figure 7C**) showed an AUROC of 0.652 (SD 0.018), which improved to 0.749 (SD 0.016) when biomarkers were included (**Figure 7D**). In the external testing cohort, the AUROC increased from 0.655 (SD 0.025) without biomarkers (**Figure 7E**) to 0.721 (SD 0.024) with biomarkers (**Figure 7F**). These results demonstrate that incorporating biomarkers significantly enhances model performance in both cohorts.

## Discussion

### Main findings

Our findings highlight that integrating biomarker measurements with clinical data enables effective prediction of future impulsivity risk (30). Univariate and multivariate logistic regression, along with time trend analyses, identified several biomarkers associated with impulsivity. These biomarkers, when combined with clinical data, significantly enhanced model performance, as demonstrated by a marked increase in AUROC and other metrics. Notably, the model yielded similar performance in both cross-validation and external cohorts, indicating good generalizability across datasets (31). Furthermore, subgroup analyses revealed a sex-specific interaction, with differences in the expression levels of key biomarkers between males and females, suggesting that sex may modulate the predictive value of these biomarkers for impulsivity risk. Results interpretation is strengthened by our study design, which ensured that biomarker and clinical data were collected at admission, before any impulsivity escalation occurred. By restricting outcomes to events within the first week post-admission, the model captures prospective risk rather than concurrent behavioral states.

Our study identifies several biomarkers that are significantly associated with future impulsivity. While UA (32), GLO (33), K (34), APOE (35), and MON (36) has been previously linked to impulsivity, biomarkers such as RBC, BAS, TBIL, HBsAb, and ALB have not been widely studied in this context. These biomarkers likely reflect underlying physiological processes, including metabolic (37) and immune system dysfunctions (38), which may contribute to the development of impulsivity. The observed temporal changes in biomarkers like UA, TBA, and K further suggest that impulsivity risk is dynamic and influenced by ongoing biological alterations (39). These findings emphasize the potential for biomarkers to serve as indicators of impulsivity risk and highlight the need for further exploration of the mechanisms linking these biomarkers to impulsivity.

CatBoost, the best-performing model, demonstrated the highest AUROC in both the cross-validation and external testing cohorts. The inclusion of biomarkers notably improved model performance, further validating the importance of biomarkers in enhancing predictive capabilities. Feature importance analysis, performed using CatBoost and SHAP, identified biomarkers such as UA, as the most influential predictors, confirming their critical role in the prediction of impulsivity (40). The SHAP heatmap analysis revealed complex, heterogeneous response patterns for biomarkers like UA, GLO, APOE, RBC, TBIL, HBsAb, and ALB, highlighting that feature interactions may be intricate and difficult to capture with traditional methods. This underscores the advantage of using machine learning techniques, which are better equipped to recognize and model such complex relationships in predictive tasks (41).

Several biomarkers exhibited significant different responses between male and female groups, and notable changes were observed over time following the occurrence of impulsivity. These differences may reflect underlying sex-specific physiological processes, such as hormonal regulation (42) and metabolic pathways (43), which could potentially modulate impulsivity risk and biomarker expression over time. Furthermore, an interaction between sex and the biomarker UA was identified, suggesting that the predictive value of UA for impulsivity risk may vary between males and females. This finding aligns with the previously discussed significant role of UA in predicting impulsivity risk (37). These results underscore the importance of considering sex differences in predictive models and suggest that further investigation into the underlying mechanisms of these biomarkers, particularly UA, is warranted.

Notably, although the model predicts impulsivity within a short time window, this prediction precedes routine clinical risk assessments and behavioral escalation, and thus provides clinically actionable information. In real-world psychiatric wards, where IBRAS or similar scales are administered weekly, our model can enable proactive identification of high-risk patients, allowing for timely implementation of targeted interventions, staffing adjustments, and personalized safety protocols. In this way, the model complements, rather than replaces, existing clinical assessments, and bridges a critical gap between admission and routine risk detection.

### Strengths and limitations

This analysis is strengthened by the use of a well-characterized, multicenter, and longitudinal real-world cohort, offering robust evidence derived from a large sample size and a rigorous study design. However, several limitations should be considered. The data were exclusively from hospitalized patients in a single city, which may limit the generalizability of the findings and introduce potential selection bias. Furthermore, missing data could introduce confounding factors, affecting the consistency and reliability of the results.

## Conclusions

This cohort study identifies a reproducible biomarker signature that is significantly correlated with future impulsivity risk in SCZ patients, enhancing the predictive accuracy and clinical utility of models based on routinely accessible patient data. Furthermore, we observed a significant correlation between sex and impulsivity risk, suggesting that sex-specific factors may influence impulsivity, which warrants further investigation into this potential relationship.

## Data Availability

The raw data supporting the conclusions of this article will be made available by the authors, without undue reservation.
