# Supplementary material for: Biomarker signatures as predictors of future impulsivity in schizophrenia: a multi-center study
Source: Front Psychiatry. 2025 Sep 29;16:1620131. doi: 10.3389/fpsyt.2025.1620131 (PMC12516360; doi:10.3389/fpsyt.2025.1620131)
Supplement: Supplementary file 1 [file DataSheet1.docx]

## Supplemental content

**Supplemental tables**

Table S1: Assessment of included features

| **Feature** | **Description** |
| --- | --- |
| ***Blood routines*** | |
| Mean corpuscular hemoglobin concentration (MCHC) | First traceable record examined from 1 week prior to admission to the day of admission.  Continuous variable, measured in g/L |
| Mean corpuscular hemoglobin (MCH) | First traceable record examined from 1 week prior to admission to the day of admission.  Continuous variable, measured in pg |
| Hemoglobin (HGB) | First traceable record examined from 1 week prior to admission to the day of admission.  Continuous variable, measured in g/L |
| Red blood cell (RBC) | First traceable record examined from 1 week prior to admission to the day of admission.  Continuous variable, measured in 10^12/L |
| Red cell distribution width coefficient of variation (RDW-CV) | First traceable record examined from 1 week prior to admission to the day of admission.  Continuous variable, measured in percentage (%) |
| Red cell distribution width standard deviation (RDW-SD) | First traceable record examined from 1 week prior to admission to the day of admission.  Continuous variable, measured in percentage (%) |
| Mean corpuscular volume (MCV) | First traceable record examined from 1 week prior to admission to the day of admission.  Continuous variable, measured in fL |
| White blood cell (WBC) | First traceable record examined from 1 week prior to admission to the day of admission.  Continuous variable, measured in 10^9/L |
| Absolute value of basophils (BAS) | First traceable record examined from 1 week prior to admission to the day of admission.  Continuous variable, measured in 10^9/L |
| Absolute value of eosinophil (EOS) | First traceable record examined from 1 week prior to admission to the day of admission.  Continuous variable, measured in 10^9/L |
| Absolute value of neutrophils (NEU) | First traceable record examined from 1 week prior to admission to the day of admission.  Continuous variable, measured in 10^9/L |
| Absolute value of monocyte (MON) | First traceable record examined from 1 week prior to admission to the day of admission.  Continuous variable, measured in 10^9/L |
| Absolute value of lymphocyte (LYM) | First traceable record examined from 1 week prior to admission to the day of admission.  Continuous variable, measured in 10^9/L |
| Platelet (PLT) | First traceable record examined from 1 week prior to admission to the day of admission.  Continuous variable, measured in 10^9/L |
| Mean platelet volume (MPV) | First traceable record examined from 1 week prior to admission to the day of admission.  Continuous variable, measured in fL |
| Mean platelet width (PDW) | First traceable record examined from 1 week prior to admission to the day of admission.  Continuous variable, measured in fL |
| Plateletcrit (PCT) | First traceable record examined from 1 week prior to admission to the day of admission.  Continuous variable, measured in percentage (%) |
| Platelet -larger cell ratio (P-LCR) | First traceable record examined from 1 week prior to admission to the day of admission.  Continuous variable, measured in percentage (%) |
| C-reactive protein (CRP) | First traceable record examined from 1 week prior to admission to the day of admission.  Continuous variable, measured in mg/L |
| ***Liver function*** | |
| Alkaline phosphatase (ALP) | First traceable record examined from 1 week prior to admission to the day of admission.  Continuous variable, measured in IU/L |
| Total bilirubin (TBIL) | First traceable record examined from 1 week prior to admission to the day of admission.  Continuous variable, measured in μmol/L |
| Total protein (TP) | First traceable record examined from 1 week prior to admission to the day of admission.  Continuous variable, measured in g/L |
| Direct bilirubin (DBIL) | First traceable record examined from 1 week prior to admission to the day of admission.  Continuous variable, measured in μmol/L |
| Total bile acid (TBA) | First traceable record examined from 1 week prior to admission to the day of admission.  Continuous variable, measured in μmol/L |
| Globulin (GLO) | First traceable record examined from 1 week prior to admission to the day of admission.  Continuous variable, measured in g/L |
| Albumin (ALB) | First traceable record examined from 1 week prior to admission to the day of admission.  Continuous variable, measured in g/L |
| Prealbumin (PALB) | First traceable record examined from 1 week prior to admission to the day of admission.  Continuous variable, measured in mg/L |
| ***Renal function*** | |
| Blood urea nitrogen (UREA) | First traceable record examined from 1 week prior to admission to the day of admission.  Continuous variable, measured in mmol/L |
| Uric acid (UA) | First traceable record examined from 1 week prior to admission to the day of admission.  Continuous variable, measured in μmol/L |
| Creatinine (CR) | First traceable record examined from 1 week prior to admission to the day of admission.  Continuous variable, measured in μmol/L |
| ***Blood lipid and glucose*** | |
| Total cholesterol (CHO) | First traceable record examined from 1 week prior to admission to the day of admission.  Continuous variable, measured in mmol/L |
| Triglyceride (TG) | First traceable record examined from 1 week prior to admission to the day of admission.  Continuous variable, measured in mmol/L |
| Lipoprotein(a) (LPA) | First traceable record examined from 1 week prior to admission to the day of admission.  Continuous variable, measured in mg/L |
| High density lipoprotein cholesterol (HDL-C) | First traceable record examined from 1 week prior to admission to the day of admission.  Continuous variable, measured in mmol/L |
| Low density lipoprotein cholesterol (LDL-C) | First traceable record examined from 1 week prior to admission to the day of admission.  Continuous variable, measured in mmol/L |
| Apolipoprotein B (APOB) | First traceable record examined from 1 week prior to admission to the day of admission.  Continuous variable, measured in g/L |
| Apolipoprotein AⅠ(APOAⅠ) | First traceable record examined from 1 week prior to admission to the day of admission.  Continuous variable, measured in g/L |
| Apolipoprotein E (APOE) | First traceable record examined from 1 week prior to admission to the day of admission.  Continuous variable, measured in mg/L |
| Fasting glucose (GLU) | First traceable record examined from 1 week prior to admission to the day of admission.  Continuous variable, measured in mmol/L |
| ***Hepatitis virus*** | |
| Hepatitis C virus (HCV) | First traceable record examined from 1 week prior to admission to the day of admission.  Continuous variable, measured in IU/mL |
| Hepatitis B virus E antibody (HBeAb) | First traceable record examined from 1 week prior to admission to the day of admission.  Continuous variable, measured in PEIU/mL |
| Hepatitis B virus E antigen (HBeAg) | First traceable record examined from 1 week prior to admission to the day of admission.  Continuous variable, measured in PEIU/mL |
| Hepatitis B virus core antibody (HBcAb) | First traceable record examined from 1 week prior to admission to the day of admission.  Continuous variable, measured in PEIU/mL |
| Hepatitis B core antibody immunoglobulin M (HBcAbIgM) | First traceable record examined from 1 week prior to admission to the day of admission.  Continuous variable, measured in PEIU/mL |
| Hepatitis B virus surface antibody (HBsAb) | First traceable record examined from 1 week prior to admission to the day of admission.  Continuous variable, measured in mIU/mL |
| Hepatitis B virus S antigen (HBsAg) | First traceable record examined from 1 week prior to admission to the day of admission.  Continuous variable, measured in mIU/mL |
| Hepatitis A virus immunoglobulin M (HAV-IgM) | First traceable record examined from 1 week prior to admission to the day of admission.  Continuous variable, measured in mIU/mL |
| ***Electrolyte*** | |
| Natrium (Na) | First traceable record examined from 1 week prior to admission to the day of admission.  Continuous variable, measured in mmol/L |
| Kalium (K) | First traceable record examined from 1 week prior to admission to the day of admission.  Continuous variable, measured in mmol/L |
| Chlorine (Cl) | First traceable record examined from 1 week prior to admission to the day of admission.  Continuous variable, measured in mmol/L |
| ***Urine routines*** | |
| SG (Specific gravity) | First traceable record examined from 1 week prior to admission to the day of admission.  Continuous variable, measured in g/mL |
| Urine glucose (UGLU) | First traceable record examined from 1 week prior to admission to the day of admission.  Categorical variable, measured in negative, ±, +, ++, +++, and ++++ |
| Urinary bilirubin (BIL) | First traceable record examined from 1 week prior to admission to the day of admission.  Categorical variable, measured in negative, ±, +, ++, and +++ |
| Urine ketones (KET) | First traceable record examined from 1 week prior to admission to the day of admission.  Categorical variable, measured in negative, ±, +, ++, and +++ |
| Urinary acidity and alkalinity (PH) | First traceable record examined from 1 week prior to admission to the day of admission.  Continuous variable, measured in acidity and alkalinity |
| Urinary nitrite (NIT) | First traceable record examined from 1 week prior to admission to the day of admission.  Categorical variable, measured in negative and positive |
| Urinary protein (PRO) | First traceable record examined from 1 week prior to admission to the day of admission.  Categorical variable, measured in mg |
| Urine white blood cell count (UWBC) | First traceable record examined from 1 week prior to admission to the day of admission.  Continuous variable, measured in number/μL |
| ***Thyroid function*** | |
| Triiodothyronine (T3) | First traceable record examined from 1 week prior to admission to the day of admission.  Continuous variable, measured in ng/mL |
| Free triiodothyronine (FT3) | First traceable record examined from 1 week prior to admission to the day of admission.  Continuous variable, measured in nmol/L |
| Thyroid stimulating hormone (TSH) | First traceable record examined from 1 week prior to admission to the day of admission.  Continuous variable, measured in uIU/mL |
| Serum free thyroxine (FT4) | First traceable record examined from 1 week prior to admission to the day of admission.  Continuous variable, measured in pmol/L |
| Tetraiodothyronine (T4) | First traceable record examined from 1 week prior to admission to the day of admission.  Continuous variable, measured in ug/dL |
| Anti-thyroglobulin antibodies (TgAb) | First traceable record examined from 1 week prior to admission to the day of admission.  Continuous variable, measured in IU/mL |
| Thyroglobulin antibodies (TPOAb) | First traceable record examined from 1 week prior to admission to the day of admission.  Continuous variable, measured in IU/mL |
| ***Gonadal hormone*** | |
| Prolactin (PRL) | First traceable record examined from 1 week prior to admission to the day of admission.  Continuous variable, measured in uIU/mL |
| ***Initial treatments*** | |
| Chlorpromazine (CPZ) | Chlorpromazine was defined by the usage of chlorpromazine that was prescribed between 30 days prior to admission and 1 day after admission that has been taken for at least 7 days and reached therapeutic dosage (≥ 200 mg/d).  Categorical variable |
| Perphenazine (PER) | Perphenazine was defined by the usage of perphenazine that was prescribed between 30 days prior to admission and 1 day after admission that has been taken for at least 7 days and reached therapeutic dosage (≥ 20 mg/d).  Categorical variable |
| Haloperidol (HAL) | Haloperidol was defined by the usage of haloperidol that was prescribed between 30 days prior to admission and 1 day after admission that has been taken for at least 7 days and reached therapeutic dosage (≥ 2 mg/d).  Categorical variable |
| Sulpiride (SUL) | Sulpiride was defined by the usage of sulpiride that was prescribed between 30 days prior to admission and 1 day after admission that has been taken for at least 7 days and reached therapeutic dosage (≥ 400 mg/d).  Categorical variable |
| Clozapine (CLOZ) | Clozapine was defined by the usage of clozapine that was prescribed between 30 days prior to admission and 1 day after admission that has been taken for at least 7 days and reached therapeutic dosage (≥ 200 mg/d).  Categorical variable |
| Olanzapine (OLA) | Olanzapine was defined by the usage of olanzapine that was prescribed between 30 days prior to admission and 1 day after admission that has been taken for at least 7 days and reached therapeutic dosage (≥ 5 mg/d).  Categorical variable |
| Risperidone (RIS) | Risperidone was defined by the usage of risperidone that was prescribed between 30 days prior to admission and 1 day after admission that has been taken for at least 7 days and reached therapeutic dosage (≥ 2 mg/d).  Categorical variable |
| Ziprasidone (ZIP) | Ziprasidone was defined by the usage of ziprasidone that was prescribed between 30 days prior to admission and 1 day after admission that has been taken for at least 7 days and reached therapeutic dosage (≥ 40 mg/d).  Categorical variable |
| Aripiprazole (ARI) | Aripiprazole was defined by the usage of aripiprazole  that was prescribed between 30 days prior to admission and 1 day after admission that has been taken for at least 7 days and reached therapeutic dosage (≥ 10 mg/d).  Categorical variable |
| Amisulpride (AMIS) | Amisulpride was defined by the usage of amisulpride  that was prescribed between 30 days prior to admission and 1 day after admission that has been taken for at least 7 days and reached therapeutic dosage (≥ 400 mg/d).  Categorical variable |
| Quetiapine Fumarate (QF) | Quetiapine Fumarate was defined by the usage of quetiapine fumarate that was prescribed between 30 days prior to admission and 1 day after admission that has been taken for at least 7 days and reached therapeutic dosage (≥ 150 mg/d).  Categorical variable |
| Paliperidone (PAL) | Paliperidone was defined by the usage of paliperidone  that was prescribed between 30 days prior to admission and 1 day after admission that has been taken for at least 7 days and reached therapeutic dosage (≥ 6 mg/d).  Categorical variable |
| Clomipramine (CLOM) | Clomipramine was defined by the usage of clomipramine that was prescribed between 30 days prior to admission and 1 day after admission that has been taken for at least 7 days and reached therapeutic dosage (≥ 100 mg/d).  Categorical variable |
| Amitriptyline (AMIT) | Amitriptyline was defined by the usage of amitriptyline that was prescribed between 30 days prior to admission and 1 day after admission that has been taken for at least 7 days and reached therapeutic dosage (≥ 100 mg/d).  Categorical variable |
| Doxepin (DOX) | Doxepin was defined by the usage of doxepin that was prescribed between 30 days prior to admission and 1 day after admission that has been taken for at least 7 days and reached therapeutic dosage (≥ 100 mg/d).  Categorical variable |
| Amoxapine (AMO) | Amoxapine was defined by the usage of amoxapine that was prescribed between 30 days prior to admission and 1 day after admission that has been taken for at least 7 days and reached therapeutic dosage (≥ 100 mg/d).  Categorical variable |
| Citalopram (CIT) | Citalopram was defined by the usage of citalopram that was prescribed between 30 days prior to admission and 1 day after admission that has been taken for at least 7 days and reached therapeutic dosage (≥ 20 mg/d).  Categorical variable |
| Escitalopram (ESC) | Escitalopram was defined by the usage of escitalopram that was prescribed between 30 days prior to admission and 1 day after admission that has been taken for at least 7 days and reached therapeutic dosage (≥ 10 mg/d).  Categorical variable |
| Sertraline (SER) | Sertraline was defined by the usage of sertraline that was prescribed between 30 days prior to admission and 1 day after admission that has been taken for at least 7 days and reached therapeutic dosage (≥ 50 mg/d).  Categorical variable |
| Fluoxetine (FLUO) | Fluoxetine was defined by the usage of fluoxetine that was prescribed between 30 days prior to admission and 1 day after admission that has been taken for at least 7 days and reached therapeutic dosage (≥ 20 mg/d).  Categorical variable |
| Paroxetine (PAR) | Paroxetine was defined by the usage of paroxetine that was prescribed between 30 days prior to admission and 1 day after admission that has been taken for at least 7 days and reached therapeutic dosage (≥ 20 mg/d).  Categorical variable |
| Fluvoxamine (FLUV) | Fluvoxamine was defined by the usage of fluvoxamine that was prescribed between 30 days prior to admission and 1 day after admission that has been taken for at least 7 days and reached therapeutic dosage (≥ 50 mg/d).  Categorical variable |
| Mirtazapine (MIR) | Mirtazapine was defined by the usage of mirtazapine that was prescribed between 30 days prior to admission and 1 day after admission that has been taken for at least 7 days and reached therapeutic dosage (≥ 30 mg/d).  Categorical variable |
| Venlafaxine (VEN) | Venlafaxine was defined by the usage of venlafaxine that was prescribed between 30 days prior to admission and 1 day after admission that has been taken for at least 7 days and reached therapeutic dosage (≥ 75 mg/d).  Categorical variable |
| Electroconvulsive Therapy (ECT) | Electroconvulsive therapy is defined as a psychiatric treatment where a generalized seizure is electrically induced to manage refractory mental disorders, which was executed between 30 days prior to admission and 1 day after admission.  Categorical variable (yes/no) |
| ***Admission information*** | |
| Sex | Recorded on the day of admission along with the first medical record.  Categorical variable, recorded as male and female |
| Age | Recorded on the day of admission along with the first medical record.  Continuous variable, measured in years. |
| Diagnosis | The primary psychiatric diagnosis recorded on the day of admission along with the first medical record.  Categorical variable, recorded as schizophrenia |
| ***Outcome measurements*** |  |
| Impulsive Behavior Risk Assessment Scale | Impulsivity was assessed by the Impulsive Behavior Risk Assessment Scale (IBRAS) made by Chinese experts combining the Modified Overt Aggression Scale (MOAS) and the Impulsivity Screening-10 (V-RISK-10), which contains 7 items, and the score of each item is 0 for no, while the score for yes varies (1, 2, 3, or 5), depending on the degree of severity of the question. A score of ≥ 5 indicates high risk, which was used as the cutoff value for this study. According to institutional requirement and clinical pathway, the risk assessment is conducted weekly with qualified nurses. |

Table S2: Study participants

| Before propensity score matching | | | | |
| --- | --- | --- | --- | --- |
|  | Control | Impulsivity | *P* value | SMD |
| Age, mean (±SD), y | 49.7 (±13.8) | 42.5 (±12.9) | <0.001***^a^ | 0.18 |
| Sex-male, number (%) | 307 (50) | 516 (58.50) | 0.001**^b^ | 0.07 |
| Medication, number (%) |  |  |  |  |
| Typical antipsychotics | 151 (24.59) | 148 (16.78) | <0.001***^b^ | 0.02 |
| Atypical antipsychotics | 571 (93) | 845 (95.80) | 0.018*^b^ | 0.04 |
| Mood stabilizer | 15 (2.44) | 25 (2.83) | 0.644^c^ | 0 |
| Antiepileptic | 83 (13.52) | 127 (14.40) | 0.629^b^ | 0.05 |
| 5-hydroxytryptamine reuptake inhibitor | 12 (1.95) | 8 (0.91) | 0.083^c^ | 0.02 |
| Other transmitter inhibitors | 4 (0.65) | 1 (0.11) | 0.076^c^ | 0.04 |
| Benzodiazepine | 213 (34.69) | 305 (34.58) | 0.965^b^ | 0.06 |
| Non- benzodiazepine | 124 (20.20) | 146 (16.55) | 0.072^b^ | 0.04 |
| ECT | 100 (16.29) | 239 (27.10) | <0.001***^b^ | 0.15 |
| After propensity score matching | | | | |
| Age, mean (±SD), y | 48.8 (±14.0) | 46.3 (±13.7) | 0.002**^a^ | 0.02 |
| Sex-male, number (%) | 279 (51.0) | 299 (54.7) | 0.249^b^ | 0.01 |
| Medication, number (%) |  |  |  |  |
| Typical antipsychotics | 120 (21.9) | 115 (21.0) | 0.712^b^ | 0.04 |
| Atypical antipsychotics | 511 (93.4) | 516 (94.3) | 0.528^b^ | 0.03 |
| Mood stabilizer | 14 (2.6) | 14 (2.6) | >0.999^c^ | 0.01 |
| Antiepileptic | 72 (13.2) | 81 (14.8) | 0.433^b^ | 0.05 |
| 5-hydroxytryptamine reuptake inhibitor | 8 (1.5) | 7 (1.3) | 0.795^c^ | 0 |
| Other transmitter inhibitors | 0 | 1 (0.1) | 0.317^c^ | 0 |
| Benzodiazepine | 183 (33.5) | 200 (36.6) | 0.281^b^ | 0.01 |
| Non- benzodiazepine | 113 (20.7) | 104 (19.0) | 0.495^b^ | 0.02 |
| ECT | 99 (18.1) | 134 (24.5) | 0.012*^b^ | 0.02 |

a, Mann-Whitney U test; b, chi-squared test with continuity correction; c,Fisher’s exact test; SD, standard deviation; SMD, standardized mean difference; SPD, standardized proportion difference; IQR, interquartile range. *, *P* < 0.05, **, *P* < 0.01, ***, *P* < 0.001.

Table S3: Coefficients of cross validated LASSO logistic regression

| Features | Coefficients |
| --- | --- |
| MCHC | -6.29e-03 |
| MCH | 0 |
| HGB | 0 |
| RBC | 1.73e-01 |
| RDW-CV | 0 |
| RDW-SD | -8.96e-04 |
| MCV | 0 |
| WBC | 0 |
| BAS | 8.07e-02 |
| EOS | -3.70e-01 |
| NEU | 0 |
| MON | 4.35e-01 |
| LYM | -1.39e-02 |
| PLT | 0 |
| MPV | 0 |
| PDW | 9.45e-02 |
| PCT | 0 |
| P-LCR | 0 |
| CRP | 0 |
| ALP | 0 |
| TBIL | 1.33e-02 |
| TP | 0 |
| DBIL | 0 |
| TBA | -2.94e-02 |
| GLO | -4.24e-02 |
| ALB | 1.18e-02 |
| PALB | 1.89e-03 |
| UREA | -1.26e-02 |
| UA | 9.34e-04 |
| CR | 0 |
| CHO | 0 |
| TG | -2.48e-02 |
| LPA | -3.81e-04 |
| HDL-C | 2.32e-01 |
| LDL-C | 0 |
| APOB | 0 |
| APOAⅠ | 0 |
| APOE | 9.40e-03 |
| HCV | 0 |
| HBeAb | 0 |
| HbeAg | 0 |
| HBcAb | 0 |
| HBsAb | 1.96e-04 |
| HBsAg | 0 |
| HAV_IgM | 0 |
| Na | 2.36e-02 |
| K | -5.10e-01 |
| Cl | 0 |
| SG | 0 |
| URO | 0 |
| PH | 0 |
| BLD | 0 |
| NIT | 2.63e-01 |
| UT | -3.40e-01 |
| UWBC | 0 |
| T3 | 0 |
| FT3 | 0 |
| FT4 | 0 |
| T4 | -1.92e-03 |
| TgAb | 0 |
| TPOAb | 0 |
| GLU | 5.37e-02 |
| UGLU (negative) | 0 |
| UGLU (+) | 0 |
| UGLU (++) | 0 |
| UGLU (+++) | 0 |
| UGLU (++++) | 0 |
| PRO (negative) | 0 |
| PRO (+) | 0 |
| PRO (++) | 1.96e-01 |
| PRO (+++) | -1.47e-01 |
| BIL (negative) | 0 |
| BIL (+) | 0 |
| BIL (++) | 1.15e-01 |
| BIL (+++) | 0 |
| KET (negative) | -3.07e-01 |
| KET (+) | 0 |
| KET (++) | 0 |
| KET (+++) | 0 |
| KET (++++) | -6.79e-01 |

# Table S4: Logistic regression and interaction effect analysis

|  | Univariate odds ratio (95% CI) | *P* value | Multivariate odds ratio (95% CI) | *P* value | Interaction with sex |
| --- | --- | --- | --- | --- | --- |
| MCHC | 0.99 (0.99-1.00) | 0.353 | 0.98 (0.97-1.00) | 0.007** | 0.810 |
| RBC | 1.60 (1.30-1.99) | < 0.001*** | 1.16 (0.86-1.57) | 0.324 | 0.880 |
| RDW_SD | 0.97 (0.94-1.00) | 0.088 | 0.98 (0.94-1.02) | 0.255 | 0.395 |
| BAS | 1.16 (1.08-1.23) | < 0.001*** | 1.14 (0.94-1.39) | 0.175 | 0.206 |
| EOS | 0.48 (0.17-1.29) | 0.149 | 0.82 (0.13-5.06) | 0.832 | 0.941 |
| MON | 3.44 (1.84-6.54) | < 0.001*** | 3.43 (0.87-13.48) | 0.077 | 0.494 |
| LYM | 1.01 (0.85-1.20) | 0.920 | 0.71 (0.43-1.18) | 0.183 | 0.858 |
| PDW | 1.15 (1.05-1.37) | 0.017* | 1.19 (1.05-1.35) | 0.006** | 0.365 |
| TBIL | 1.04 (1.02-1.06) | < 0.001*** | 1.02 (1.00-1.04) | 0.051 | 0.424 |
| TBA | 0.95 (0.92-0.98) | < 0.001*** | 0.96 (0.93-0.99) | 0.024* | 0.109 |
| GLO | 0.96 (0.93-0.99) | 0.004** | 0.94 (0.90-0.97) | *<* 0.001*** | 0.826 |
| ALB | 1.08 (1.04-1.12) | < 0.001* | 1.00 (1.00-1.01) | 0.960 | 0.704 |
| PALB | 1.00 (1.00-1.01) | 0.001** | 1.00 (1.00-1.01) | *<* 0.001*** | 0.962 |
| UREA | 0.97 (0.92-1.02) | 0.283 | 0.95 (0.88-1.02) | 0.165 | 0.846 |
| UA | 1.00 (1.00-1.00) | < 0.001*** | 1.00 (1.00-1.01) | 0.013* | 0.012* |
| TG | 0.95 (0.84-1.06) | 0.392 | 0.80 (0.64-1.00) | 0.047* | 0.343 |
| LPA | 0.99 (0.99-1.00) | 0.031* | 1.00 (0.99-1.00) | 0.075 | 0.150 |
| HDL_C | 1.07 (0.99-1.17) | 0.11 | 1.14 (0.69-1.89) | 0.611 | 0.793 |
| APOE | 1.06 (1.00-1.12) | 0.028* | 1.12 (0.98-1.28) | 0.109 | 0.175 |
| HBsAb | 1.00 (1.00-1.00) | 0.007** | 1.00 (1.00-1.01) | 0.014* | 0.316 |
| Na | 1.05 (1.01-1.10) | 0.009** | 1.06 (1.01-1.11) | 0.031* | 0.390 |
| K | 0.54 (0.39-0.75) | < 0.001*** | 0.54 (0.37-1.07) | 0.002** | 0.296 |
| NIT | 1.39 (0.95-2.07) | 0.095 | 1.71 (1.11-2.64) | 0.016* | 0.356 |
| UT | 0.54 (0.28-1.02) | 0.063 | 0.50 (0.24-1.07) | 0.073 | 0.385 |
| T4 | 0.99 (0.99-1.00) | 0.501 | 0.99 (0.99-1.00) | 0.004** | 0.599 |
| GLU | 1.06 (1.00-1.12) | 0.029* | 1.14 (1.06-1.22) | *<* 0.001*** | 0.094 |
| BIL |  |  |  |  |  |
| ++ | 3.02 (0.69-20.69) | 0.177 | 2.00 (0.33-12.26) | 0.453 | 0.366 |
| KET |  |  |  |  |  |
| negative | 0.57 (0.43-0.76) | < 0.001*** | 0.64 (0.45-0.91) | 0.012** | 0.758 |
| ++++ | 0.54 (0.19-1.43) | 0.228 | 0.23 (0.07-0.75) | 0.015* | 0.339 |
| PRO |  |  |  |  |  |
| ++ | 1.63 (0.85-3.20) | 0.146 | 1.77 (0.83-3.75) | 0.139 | 0.544 |
| +++ | 0.33 (0.05-1.44) | 0.177 | 0.40 (0.06-2.86) | 0.360 | 0.361 |

*, two-sided *P* value < 0.05; **, two-sided *P* value < 0.01; ***, two-sided *P* value < 0.001.

Table S5: Changes in biomarker signatures

|  | Baseline | After | Mean difference | *P* value |
| --- | --- | --- | --- | --- |
| MCHC, median (IQR), g/L | 333 (326-339) | 332 (326-339) | 0.45 | 0.945^a^ |
| RBC, median (IQR),10^^12^/L | 4.49 (4.17-4.88) | 4.41 (4.09-4.74) | -0.11 | 0.01*^a^ |
| BAS, median (IQR),10^^9^/L | 0.03 (0.02-0.04) | 0.02 (0.01-0.03) | -0.01 | *<* 0.001***^a^ |
| EOS, median (IQR),10^^9^/L | 0.09 (0.04-0.16) | 0.1 (0.05-0.18) | 0.02 | 0.014*^a^ |
| MON, median (IQR),10^^9^/L | 0.5 (0.38-0.65) | 0.4 (0.32-0.52) | -0.09 | *<* 0.001***^a^ |
| LYM, median (IQR),10^^9^/L | 1.85 (1.39-2.29) | 1.66 (1.35-2.13) | -0.13 | 0.007**^a^ |
| PDW, median (IQR), fl | 0.12 (0.11-0.14) | 0.12 (0.11-0.13) | 0.09 | 0.375^a^ |
| TBIL, median (IQR), umol/L | 14.6 (11-20) | 11.9 (9.2-14.98) | -3.94 | *<* 0.001***^a^ |
| TBA, median (IQR), umol/L | 3 (1.8-5) | 3.8 (2.3-5.7) | 0.86 | *<* 0.001***^a^ |
| GLO, median (IQR), g/L | 24.4 (22.1-26.9) | 24.7 (22.95-26.98) | 0.16 | 0.306^a^ |
| ALB, median  (IQR), g/L | 42 (40.1-44) | 42 (40.2-43.6) | -0.08 | 0.691^a^ |
| PALB, median (IQR), mg/L | 254 (207-293.5) | 270 (242.3-307.8) | 22.11 | *<* 0.001***^a^ |
| UREA, median (IQR), mmol/L | 4.62 (3.51-5.65) | 4.13 (3.43-4.91) | -0.46 | *<* 0.001***^a^ |
| UA, median (IQR), μmol/L | 355.1 (280.1-483.6) | 310 (261.6-376.5) | -45.98 | *<* 0.001***^a^ |
| TG, median (IQR), IU/mL | 1.1 (0.815-1.545) | 1.32 (1.02-1.75) | 0.22 | *<* 0.001***^a^ |
| LPA, median (IQR), mg/L | 127 (66-261.5) | 139 (74.25-241.5) | 12.34 | 0.271^a^ |
| APOE, median (IQR), mg/L | 3.94 (3.32-4.79) | 3.78 (3.34-4.23) | -0.11 | 0.009**^a^ |
| Na, median (IQR),mmol/L | 142.3 (140.4-144.2) | 143 (141-144.5) | 0.54 | 0.013*^a^ |
| K, median (IQR),mmol/L | 3.8 (3.52-4.04) | 3.9 (3.7-4.1) | 0.12 | *<* 0.001***^a^ |
| T4, median (IQR),μg/dL | 10 (7.4-116) | 79.8 (6.9-106.7) | -1.12 | 0.156^a^ |
| GLU, median (IQR),mmol/L | 5.22 (4.7-6.1) | 5.09 (4.72-5.96) | -0.34 | 0.322^a^ |
| NIT (+), number (%) | 66 (12.07%) | 13 (14.44%) | 0.02 | 0.644^b^ |
| UT (+), number (%) | 15 (2.74%) | 3 (3.33%) | 0.01 | > 0.999^c^ |
| BIL (++), number (%) | 6 (1.10%) | 0 | -0.01 | 0.682^c^ |
| KET-negative, number (%) | 396 (72.53%) | 79 (87.78%) | 0.15 | 0.003**^b^ |
| KET (++++), number (%) | 6 (1.10%) | 1 (1.11%) | 0 | > 0.999^c^ |
| PRO (++), number (%) | 24 (4.40%) | 5 (5.56%) | 0.01 | 0.826^c^ |
| PRO (+++), number (%) | 2 (0.37%) | 0 | 0 | > 0.999^c^ |

a, Mann-Whitney U test; b, chi-squared test with continuity correction; c, Fisher’s exact test; IQR, interquartile range; *, two-sided *P* value < 0.05; **, two-sided *P* value < 0.01; ***, two-sided *P* value < 0.001

**Supplemental figures**

Figure S1: Standardized mean differences


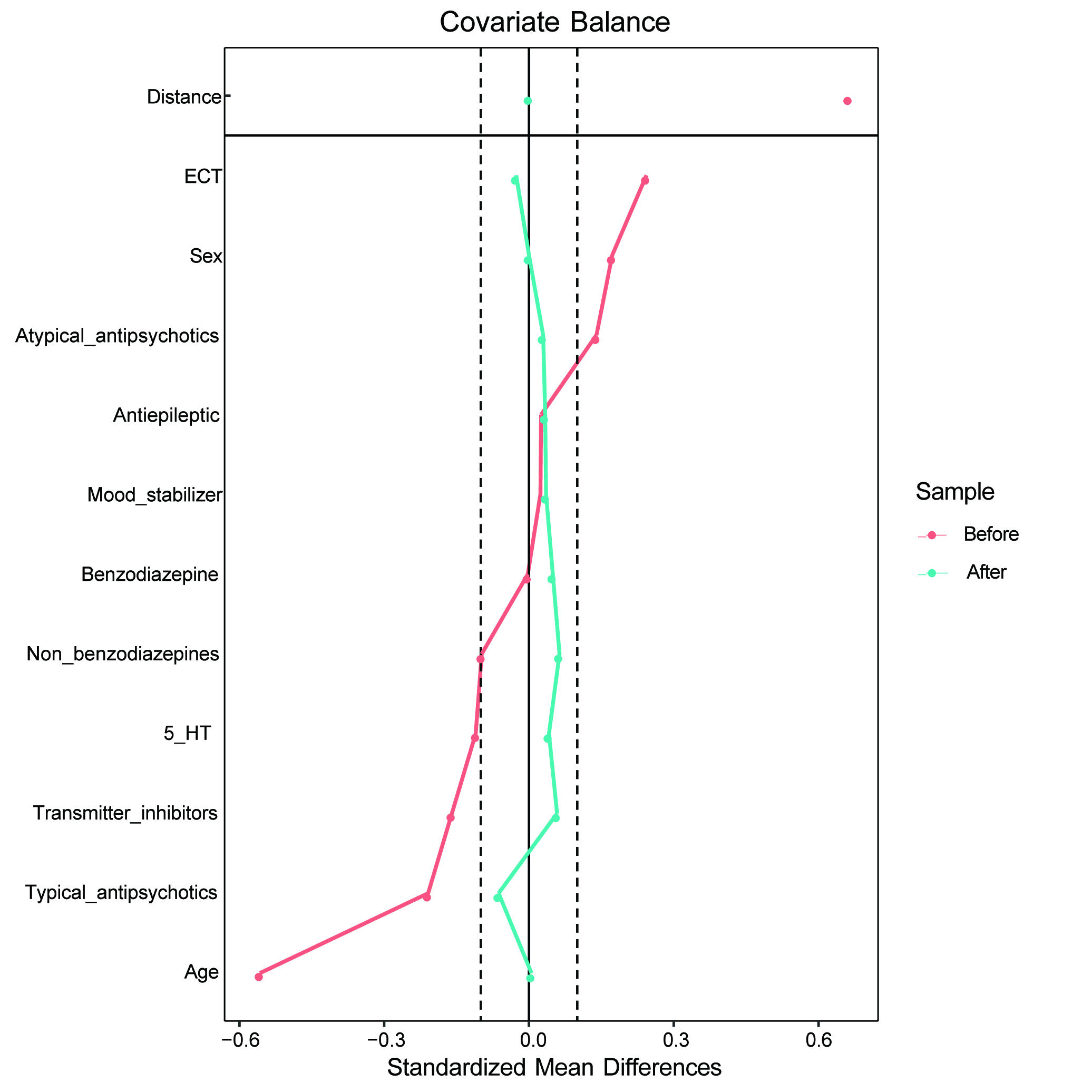
Compared to before propensity score matching, the effectiveness of the distribution of SMD values after propensity score matching has significantly improved.

Figure S2: The occurrence rate of impulsivity.

By comparing the impulsivity incidence rates between genders in the overall data, there is a statistically significant difference in impulsivity incidence rates between genders, with males showing significantly higher impulsivity incidence rates.

Figure S3: Average gain of the ten most important variables and SHAP summary plot.**
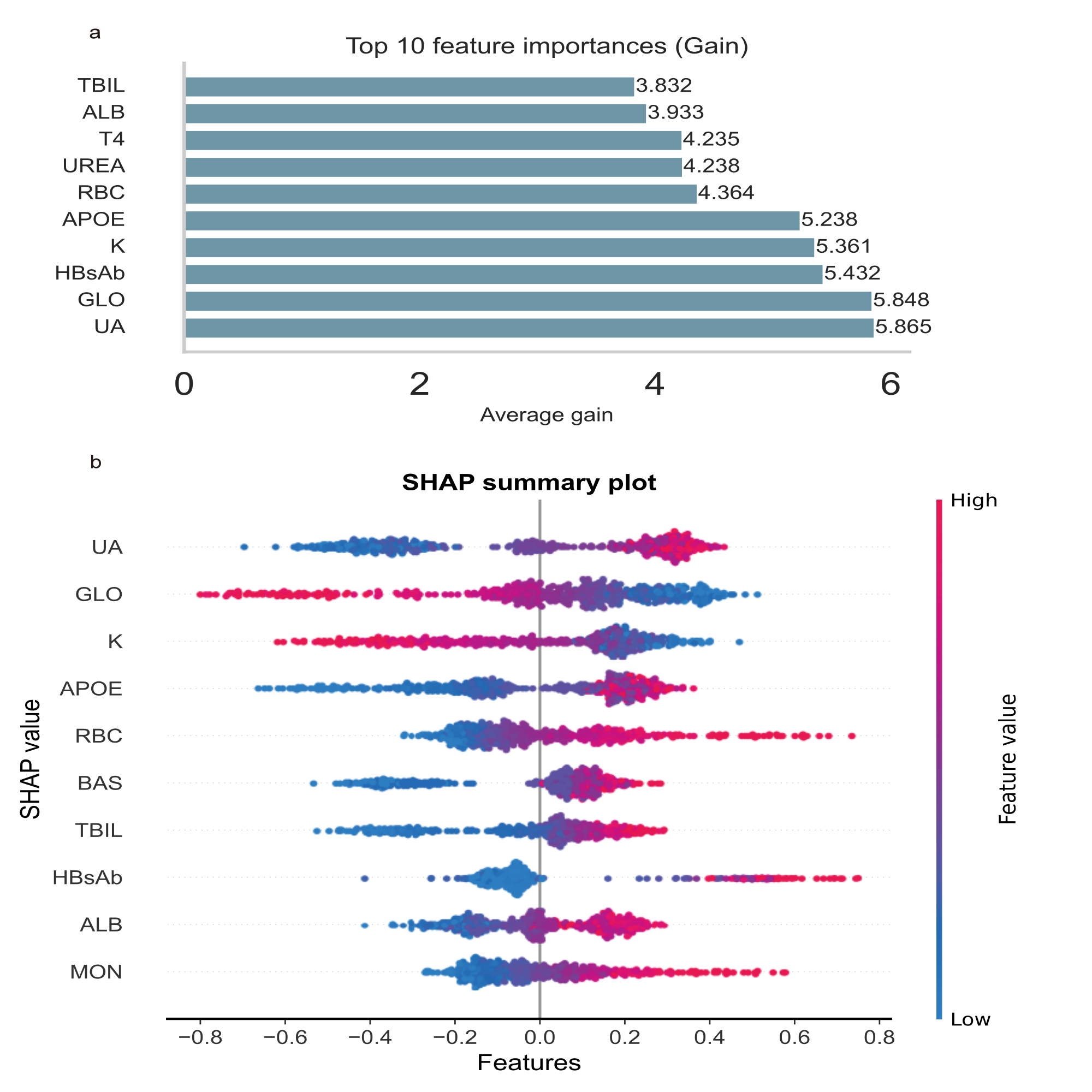
**

**a**: Average gain of the ten most important variables. **b**: SHAP summary plot. TBIL, total bilirubin; ALB, albumin; T4, tetraiodothyronine; UREA, urea nitrogen; RBC, red blood cell; APOE, apolipoprotein E; K, kalium; HBsAb, hepatitis B virus surface antibody; GLO, globulin; UA, uric acid; BAS, basophils; MON, monocyte.

Figure S4: The silhouette score.


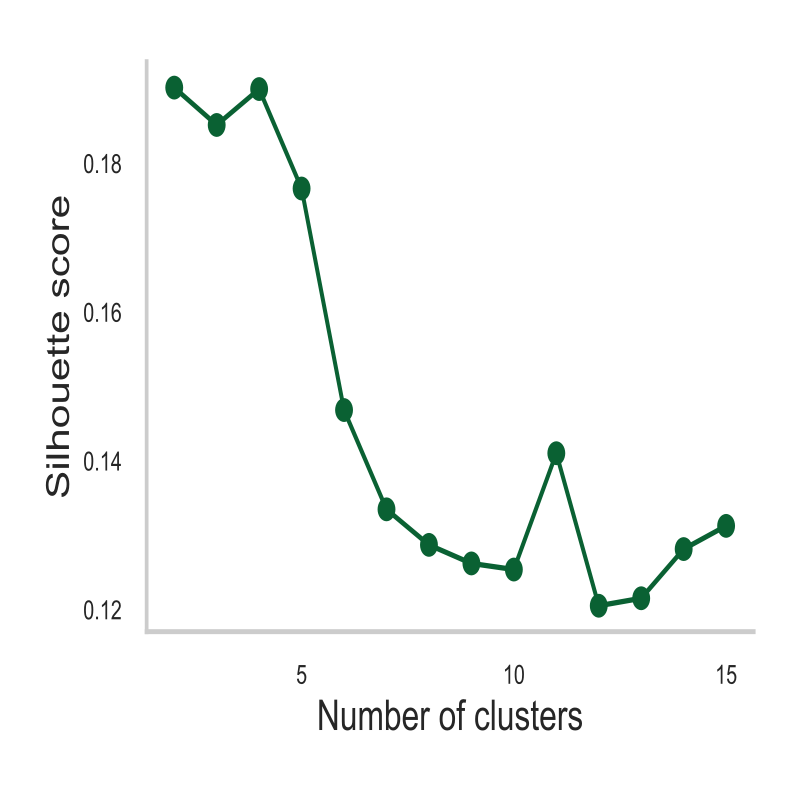
The silhouette score calculates the cohesion and separation of clusters by measuring how similar a sample is to its own cluster compared to other clusters, where a better score is obtained when there are two clusters.
